# The Biology and Genomics of Human Hair Follicles: A Focus on Androgenetic Alopecia

**DOI:** 10.3390/ijms25052542

**Published:** 2024-02-22

**Authors:** Raquel Cuevas-Diaz Duran, Emmanuel Martinez-Ledesma, Melissa Garcia-Garcia, Denisse Bajo Gauzin, Andrea Sarro-Ramírez, Carolina Gonzalez-Carrillo, Denise Rodríguez-Sardin, Alejandro Fuentes, Alejandro Cardenas-Lopez

**Affiliations:** 1 Tecnologico de Monterrey, Escuela de Medicina y Ciencias de la Salud, Monterrey 64710, NL, Mexico; 2 CapilarFix^®^, Monterrey 66220, NL, Mexicobajogauzin@gmail.com (D.B.G.);; 3 Tecnologico de Monterrey, Institute for Obesity Research, Monterrey 64849, NL, Mexico; 4 NeoMedics^®^, Monterrey 66220, NL, Mexico

**Keywords:** androgenetic alopecia, transcriptomics, genomics

## Abstract

Androgenetic alopecia is a highly prevalent condition mainly affecting men. This complex trait is related to aging and genetics; however, multiple other factors, for example, lifestyle, are also involved. Despite its prevalence, the underlying biology of androgenetic alopecia remains elusive, and thus advances in its treatment have been hindered. Herein, we review the functional anatomy of hair follicles and the cell signaling events that play a role in follicle cycling. We also discuss the pathology of androgenetic alopecia and the known molecular mechanisms underlying this condition. Additionally, we describe studies comparing the transcriptional differences in hair follicles between balding and non-balding scalp regions. Given the genetic contribution, we also discuss the most significant risk variants found to be associated with androgenetic alopecia. A more comprehensive understanding of this pathology may be generated through using multi-omics approaches.

## 1. Introduction

Androgenetic alopecia (AGA), also known as male pattern baldness, is the most prevalent hair-loss disorder in men. It has been estimated that 50% of 50-year-old men and up to 70% of older Caucasian men present AGA symptoms [1]. The prevalence and severity of AGA in other ethnicities, for example, Asian and African, have been reported to be lower than in Caucasians [2,3,4]. Furthermore, racial and age-related differences in hair loss pattern and incidence of AGA have also been demonstrated [4]. However, the incidence, age of onset, and severity of the disease are increasing across all races. AGA’s main physiologic representation is the progressive miniaturization of terminal hair follicles, eventually leading to their transformation into vellus and their atrophy [5]. Furthermore, accumulating studies report associations of AGA with other important pathologies, such as coronary heart disease, hypertension, hyperinsulinemia, obesity, and prostatic cancer, suggesting a common underlying biology [6,7,8,9,10]. The high prevalence of AGA and its potential comorbidities underscore the importance of elucidating the molecular and genetic mechanisms underlying this pathology. However, our understanding of hair follicle biology and pathology remains incomplete due to technical limitations. Hitherto, only two drugs and one device have been approved by the Food and Drug Administration (FDA). Advances in single-cell sequencing technologies and epigenomics have the potential to improve our understanding of hair follicles and unveil novel therapeutic targets.

Hair follicles (HFs) are the only mammalian organs with the ability to regenerate throughout adult life, mimicking embryonic growth. This regeneration ability is tightly regulated by a “hair clock” consisting of epithelial–mesenchymal interactions [11]. Interestingly, these cues are generated by cells within the HF itself and they drive cyclic transitions between stages of rapid growth (anagen), involution (catagen), and relative quiescence (telogen) [12]. Systemic factors also influence hair cycling. Thus, one of the main challenges of hair research is finding the underlying molecular signals that orchestrate the HF’s cycling since numerous hair pathologies are derived from an altered hair cycling.

While the perfect combination for AGA pathology is a genetic predisposition and the presence of androgens, practice in the clinic has demonstrated that blocking androgens does not always result in the reversal of miniaturized HFs. All evidence demonstrates that AGA is a multifactorial disorder mainly caused by the dysregulation of the hair cycle due to alterations between the crosstalk of numerous cell subpopulations. A more comprehensive understanding of AGA at the molecular level is needed. Herein, we will review the anatomy and the cycling of the HF focusing on the underlying cell subpopulations and their known interactions. We will also discuss the pathology of AGA and the molecular mechanisms known to contribute to this disorder. Furthermore, we will describe the studies that have been performed aimed at understanding the transcriptomic differences between human HFs obtained from balding and non-balding scalp regions. Finally, we will discuss some of the risk variants found with significant association with AGA. Studying AGA using a multi-omics approach will help resolve all the unknowns that still remain.

## 2. Functional Hair Follicle Anatomy

**Figure 1:**
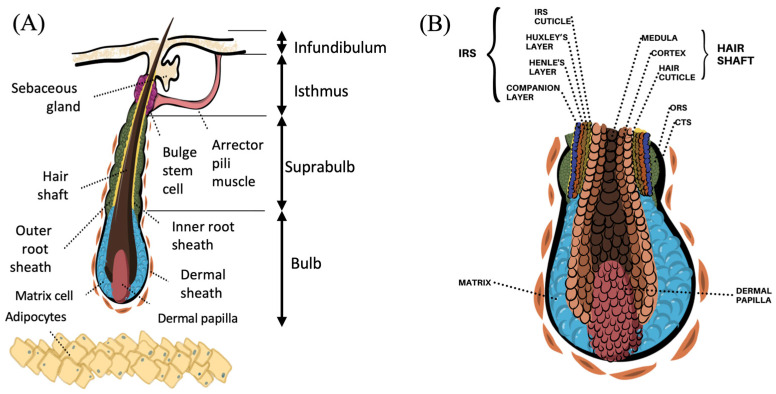
Schematic representations of the anatomy of the hair follicle. (**A**) Four main regions divide the hair follicle: bulb, suprabulb, isthmus, and infundibulum. (**B**) In a transversal view, the hair follicle appears as a cylinder formed by eight concentric layers forming the outer root sheath (ORS), inner root sheath (IRS), and the hair shaft. The IRS is composed of four layers: companion layer, Henle’s layer, Huxley’s layer, and IRS cuticle. The hair shaft is composed of the medulla, cortex, and hair cuticle.

The HF is a complex and dynamic mini-organ that comprises cells of epithelial and mesenchymal origin. The HF and its associated structures, the sebaceous gland and the arrector pili muscle, form the pilosebaceous unit. Anatomically, HFs can be divided into four regions: bulb, suprabulb, isthmus, and infundibulum segments (Figure 1A). The infundibulum is the uppermost section, beginning at the surface of the epidermis forming the hair canal and extending to the opening of the sebaceous duct. This funnel-shaped structure is filled with sebum produced in the sebaceous gland. The infundibulum serves as a reservoir and provides a penetration pathway through which topically applied compounds may interact with hair follicle cell subpopulations [13,14]. The middle section is the isthmus, the region between the sebaceous duct and the bulge. This region is marked by the insertion of the arrector pili muscle. In the lower isthmus lies the bulge, a region of scientific interest because it harbors epithelial and melanocytic stem cells. These hair follicle stem cells (HFSCs) are multipotent and quiescent, and they are involved in hair regrowth [15,16]. HFSCs in the bulge were first identified through a method that labels slow-cycling cells, an important property of stem cells, and they were referred to as “label-retaining cells” (LRCs) [17,18]. Furthermore, human and mouse bulge cells have demonstrated a high colony-forming efficiency, pinpointing their high proliferative potential [19,20,21]. Assays involving laser capture microdissection allowed the purification of bulge stem cells and the identification of specific cell surface markers. Pioneering work by Lyle et al. reported that human bulge HFSCs expressed Keratin 15 (KRT15), an intracellular intermediate filament protein [22]. However, this marker is also lowly expressed in the lower follicle, and thus other markers had to be studied. Ohyama et al. isolated human HFSCs as LRCs and profiled them using microarrays [19]. Their work allowed the identification of a panel of markers specific to human bulge HFSCs, of which CD200 stands out [19]. Expression of CD200 has also been identified in the secondary hair germ (SHG) [23]. Studies in mice demonstrated a high expression of the surface protein CD34 in bulge HFSCs [24]; however, this marker was not found in human bulge HFSCs [19], confirming biological differences between species. In human HFs, the expression of CD34 has been found in cells below the bulge in the anagen stage [25]. CD34^+^ cells proliferate in vitro, forming colonies, and they undergo apoptosis at the end of anagen; thus, they are considered progenitors of bulge HFSCs [25,26].

Upon activation at the early anagen phase, HFSCs migrate downwards along the outer root sheath (ORS) to the hair bulb matrix, where they proliferate to form the SHG [27,28]. The SHG is a transitory structure that forms in the telogen phase in the lower part of the HF, in direct contact with the dermal papilla (DP). Interestingly, numerous studies have demonstrated the role of SHG cells in anagen induction, maintenance of the HFSC niche, and in the restoration of HFSCs after injury [27,29,30,31,32,33]. SHG cells are “germinative cells” that transition to matrix progenitors (also referred to as matrix transient amplifying cells) and differentiate upon signaling from the DP to form the ascending part of the HF: the inner root sheath (IRS) and the hair shaft [34]. Importantly, SHG cells have been identified and characterized only in mouse HFs, although the hypothesis of human analogs is widely accepted [35,36]. The appearance of SHG cells in the telogen phase has hindered their characterization in human HFs since their cycling is asynchronous with long anagen and very short telogen phases compared to mice.

The bulb is the lowermost section of the HF. The bulb is separated from the bulge by a suprabulbar stretch of HF epithelium (Figure 1A). The bulb embraces the DP and the hair matrix. The bulb is considered the “hair factory” because it contains the matrix keratinocytes, a population of cells with the highest proliferation rate in the human body and with the ability to differentiate [37]. Even though matrix cells originate from the SHG, they terminally differentiate into IRS or hair shaft according to their spatial location in the matrix. The number of matrix keratinocytes determines the diameter of the hair shaft and the size of the hair bulb. Furthermore, melanocytes, derived from bulge melanocyte stem cells, reside among matrix cells. These cells phagocytose melanin or pheomelanin, proteins responsible for hair pigmentation. The DP consists of a small cluster of tightly packed fibroblasts derived from the mesenchyme. The DP is considered the “command center” since it provides key signals that regulate the timing and type of HF formed throughout adult life [38,39,40].

Transversely, the HF appears as a cylinder formed by eight concentric layers including the ORS, IRS (companion layer, Henle’s layer, Huxley’s layer, IRS cuticle), and the hair shaft (medulla, cortex, hair cuticle) (Figure 1B). Interestingly, the companion layer cells allow the upwards displacement of the IRS while being tightly connected to Henle’s layer, thus acting as a slippage plane. Each layer is characterized by the expression of specific keratins [41]. The hair shaft is composed of terminally differentiated keratinocytes compacted into a fiber of amazing tensile strength.

## 3. Hair Follicle Cycling

The HF has the unique ability to cycle through phases of active regeneration (anagen), apoptotic involution (catagen), and rest (telogen) throughout the lifetime of mammals. This cycling activity is tightly regulated by sophisticated epithelial–mesenchymal interactions which generate gradients of molecules acting as inhibitors or activators. In humans, HF cycling becomes asynchronous after birth, in contrast to mice, whose HFs are highly synchronized [11]. At any given time, approximately 80–90% of the human scalp HFs are in the anagen phase, whereas only 1–2% and 5–15% reside in the catagen or telogen phases, respectively [12,42]. The duration of each phase varies according to numerous factors, for example, anatomical location of the HF, nutritional and hormonal status, and age [43]. In the same healthy individual, the lengths of anagen and catagen phases are similar in all cycles; however, the telogen phase becomes increasingly longer with each additional cycle (correlated to aging) [44]. Human scalps have approximately 80,000–150,000 HFs and each of them undergo between 10 and 30 cycles of regeneration in a lifetime [11]. The recurrent involution and regeneration are driven by molecular mechanisms similar to those observed during fetal HF morphogenesis [45]. Furthermore, transplantation experiments have demonstrated that the cycling system is most likely located within the HF itself since this organ maintains its original cycling behavior even after being relocated [46,47]. However, experiments by Plikus et al. showed that the hair growth patterns of autologous transplanted skin in mice depicted intrinsic cycling but gradually acquired the host’s pattern, suggesting a systemic influence [48]. The exact signaling mechanisms and the clock that controls the cycling of HFs have not been completely elucidated.

### 3.1. Anagen Phase

Researchers have demonstrated that in the late telogen phase, just before anagen, cells of the SHG and DP become transcriptionally active and potentially synthesize proteins required for the telogen to anagen transition [33]. These observations suggest that signaling between DP and SHG cells is responsible for anagen induction. Studies with animal models have allowed the identification of the molecular events potentially involved in hair cycling [49,50]. Two molecules have been identified as important for HF development and cycling: insulin-like growth factor 1 (IGF1) and fibroblast growth factor 7 (FGF7). These are secreted by DP cells and they bind receptors in matrix keratinocytes [43,49]. Other signaling molecules have been found to be important for anagen induction and duration, for example, members of the TGF-β, BMP, WNT, sonic hedgehog, homeobox, and neurotrophin families [43,44,51,52].

In the laboratory, researchers have long been able to induce anagen initiation by trauma or wounding procedures, for example, through chemical exposure to depilatory agents, vigorous shaving, and hair plucking [53,54]. These procedures trigger the secretion of inflammatory cytokines and initiate anagen hair growth. Interestingly, research has demonstrated that there is a threshold for the traumatic stimulus applied and that the inflammatory response localizes to the wounding area [55]. Furthermore, anagen has been induced by numerous drugs, for example, minoxidil, cyclosporin A, FK506, norepinephrine-depleting agents, estrogen receptor antagonists, tretinoin, tumor promoting agents (TPAs), keratinocyte growth factor (KGF), hepatocyte growth factor (HGF), substance P, and capsaisin [50]. However, their complete molecular mechanisms of action remain elusive.

**Figure 2:**
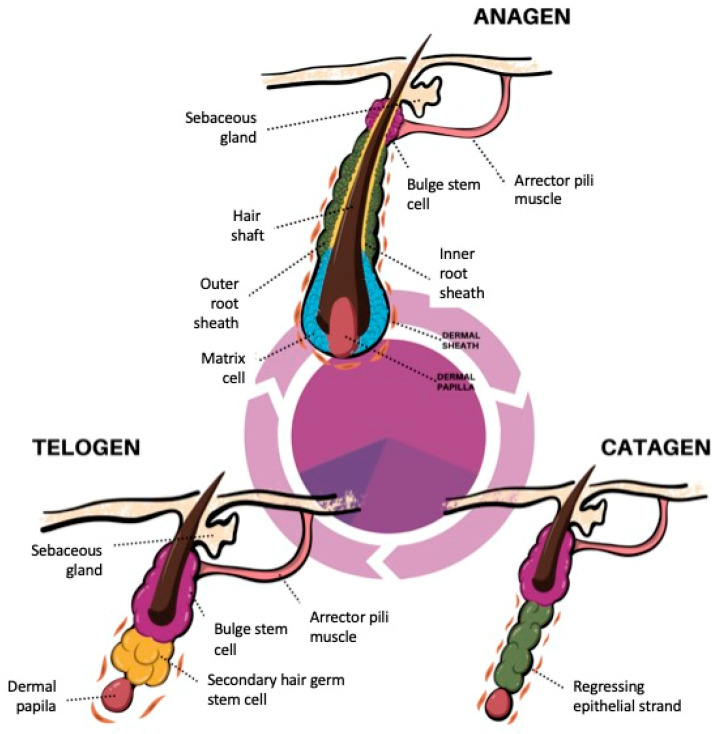
Key stages of the hair cycle. The hair follicle cycles through three phases: anagen (active regeneration), catagen (apoptotic involution), and telogen (resting phase) throughout life. In the anagen phase, the follicle becomes elongated and acquires an onion-like shape. Secondary hair germ cells (SHG) in the matrix proliferate intensively and differentiate into cells of the inner root sheath and hair shaft. After hair follicle maturation, the regression phase is initiated. Apoptosis of cells of the lower, cycling portion of the hair follicle occurs, bringing the dermal papilla into close proximity to the bulge, a condition that is maintained in the telogen phase. Molecular signaling between stem cells in the bulge (hair follicle stem cells, HFSCs) and the dermal papilla are responsible for the re-entrance into the anagen phase.

The anagen phase comprises the complete regeneration of the lower, cycling portion of the HF. It is characterized by intensive proliferation of SHG cells in the matrix and their further differentiation into epithelial hair lineages (IRS and hair shaft) [33]. Coupled to hair shaft synthesis is its pigmentation, which is performed by melanocytes only in anagen. Melanocyte stem cells residing in the bulge region proliferate and differentiate into melanocytes at the onset of anagen and migrate to the bulb matrix, where they secrete melanin pigment in the form of granules [56]. These granules are transferred to keratinocytes in the cortex and medulla where they become part of the pigmented shaft. Melanogenic activity is stringently controlled; it is switched off in catagen and remains absent in telogen [57]. Thus, the HF pigmentary unit undergoes cyclic reconstructions coupled to HF regeneration. It has been reported that the optimal reconstruction of the scalp HFs occurs only in the first 10 cycles, accounting for approximately 40 years of age. Afterwards, there is a genetically controlled exhaustion of the pigmentary potential of melanocytes, leading to hair graying [58]. In the anagen phase, the bulge progressively moves away from the DP allowing HFSCs to return to their quiescent state (Figure 2). The energetic requirement of hair shaft regeneration is high, potentially inducing angiogenesis in the HF during anagen development [59]. The anagen growth phase can last between 2 and 8 years [12]. The progression of anagen requires the continued activation of the WNT/β-catenin signaling in the DP [40]. By the end of anagen, the proliferation of cells in the matrix decreases. The cessation of this phase is controlled by fibroblast growth factor 5 (FGF5) since mice lacking this protein depict abnormally long hair (angora phenotype) as a result of an extended anagen stage [60]. Also, epidermal growth factor receptors have been found to be involved in the termination of anagen [61,62].

### 3.2. Catagen Phase

During catagen, proliferation stops, and apoptosis takes place in the keratinocytes of the matrix, IRS, and ORS, causing a rapid regression of the lower parts of the HF. Follicular melanogenesis is also stopped and some melanocytes undergo apoptosis [63]. By the end of catagen, the lower part of the HF shrinks and withdraws as an epithelial strand and the DP condenses (potentially due to loss of extracellular matrix) and moves upward proximal to the bulge. Failure of the DP in reaching the bulb may be caused by mutations in the *HAIRLESS* (*HR*) gene, resulting in permanent hair loss [64,65]. The *HR* gene codes for a transcription factor that acts as nuclear corepressor [66]. Mutations in this gene have been found both in both mice and humans, resulting in alopecia universalis and popular atrichia [64,67,68], conditions where hair never regenerates after the first shedding.

Diverse signals have been proposed as catagen-promoting agents, for example, inhibiting IGF1 receptor, TFG-β1, and hepatocyte growth factor (HGF) and activating neurotrophins (NT-3, NT-4) and BDNF [69,70,71,72,73,74]. Also, it has been demonstrated that severe stress, dexamethasone, environmental factors, trauma, and hormones like ACTH and 17β-estradiol can induce an early catagen phase [75,76,77,78]. However, the exact molecular mechanisms that trigger the catagen phase remain unknown. The catagen phase lasts 1–3 weeks leaving only club hair (a hair filament that has stopped growing but remains attached in the HF) surrounded by an epithelial cap.

### 3.3. Telogen Phase

The telogen phase was initially described as a “resting phase”; however, it coincides with major gene activity changes [79]. Genes coding for important hair cycle regulators (e.g., estrogen receptors) depict their maximum expression during telogen, suggesting that this phase is critical for the control of hair cycling [80,81]. This phase has been divided into two functional stages: refractory and competent. In the refractory telogen phase, there is an upregulation and activation of BMP2/4, thus inhibiting hair growth. Researchers have demonstrated that BMPs secreted from subcutaneous fat are able to maintain HFs in the refractory telogen phase [48]. Contrastingly, in the competent telogen phase, BMP signaling is inactivated, while WNT/β-catenin is upregulated, rendering bulge HFSCs highly sensible to anagen-inducing factors [48]. During the telogen phase, the DP lies directly below the bulge, allowing for signaling between HFSCs and DP cells. These interactions are essential for triggering a new hair cycle. The telogen phase generally lasts between 2 and 3 months before the HF re-enters the anagen phase.

## 4. Androgenetic Alopecia (AGA)

AGA, or male pattern baldness, is a multifactorial disorder caused by genetic factors, hormonal dysregulation, environmental and systemic factors, and aging. Women can also be affected by AGA, in this case referred to as female-pattern hair loss. Prevalence data in women are more variable; however, a study by Norwood reported that 6% of women under 50 and 30–40% of women older than 70 years were diagnosed with female AGA [82]. Hair loss in AGA is the result of a progressive shortening of the anagen phase resulting in miniaturization of the HFs and eventual atrophy [83]. This miniaturization is associated to abnormalities in the DP since its volume determines the hair shaft’s diameter and is correlated with the duration of the anagen phase [84]. In AGA, genetically predisposed HFs undergo a transformation from large thick pigmented hairs (terminal) to barely visible depigmented hairs (vellus) [84]. Female AGA is characterized by diffuse thinning of the vertex maintaining an intact frontal hairline. The Ludwig scale describes the patterns of diffuse scalp alopecia commonly seen in women [85]. Contrastingly, in men, hair loss follows a specific pattern, described by the scales of Hamilton [86] and Norwood [1]. The regions preferentially affected by AGA are the temples, the vertex, and the mid-frontal scalp, whereas the occipital and temporal regions are spared. This hair loss distribution is related to the density of androgen receptors (ARs) in the DP cells [87]. The success of hair transplantation techniques relies on the observation that HFs harvested from the occipital and temporal regions maintain their androgen-independence behavior even after placed in affected regions.

Androgens are potent sex hormones and known mediators of hair growth throughout the body. However, their effects are paradoxical. Upon sexual maturity, androgens increase the size of HFs acting through ARs on DP cells residing in androgen-sensitive areas (e.g., axilla, pubis, face, chest, and extremities) [84]. However, at a later age, the effect of androgens on genetically predisposed HFs is detrimental, promoting their miniaturization. Notwithstanding, the role of androgens in the development of AGA has been well established. Testosterone and its more potent and active metabolite dihydrotestosterone (DHT) circulate in the blood and bind ARs in DP cells. ARs are nuclear receptors that upon ligand binding undergo conformational changes, become activated, and bind hormone response elements in the DNA, regulating their transcription [88]. Furthermore, the enzyme 5α-reductase (type 1 and 2) mediates the conversion of testosterone to its more potent metabolite DHT in the HFs. Interestingly, individuals with genetic predisposition exhibit DP cells (within balding regions) with a higher density of ARs and an increased activity of 5α-reductase type 2 [87,89]. This combination results in a local sustained binding of DHT to ARs in DP cells, leading to hair cycle disruption. Moreover, these balding DP cells exhibit signs of premature senescence (loss of replicative potential, decrease in molecular markers), lose their ability to promote HFSC proliferation, and secrete inhibitory factors [90,91,92]. For example, balding DP cells have an increased expression of DKK1, a negative regulator of WNT signaling (critical pathway involved in anagen entry and progression) [93]. Additionally, balding DP cells secrete inflammatory cytokines such as IL-6, which inhibit anagen entry [92]. Overall, paracrine and autocrine signaling cascades triggered by androgens in genetically affected DP cells are the main cause of AGA; however, they are not fully understood.

Disruption in HFSC activation has also been demonstrated in AGA pathogenesis. Studies involving samples obtained from balding and non-balding scalp regions from AGA patients showed that bulge HFSC numbers, assessed by the expression of KRT15 and ITGA6, were maintained in balding regions; however, progenitor subpopulations CD200^+^ and CD34^+^ were decreased [23]. These observations suggest that in AGA, the conversion of HFSCs to progenitor subpopulations is decreased, affecting the number of SHG and bulb matrix cells that regenerate the hair shaft, therefore resulting in miniaturized hair. Advances in single-cell RNA sequencing are allowing the study of the heterogeneity of HF progenitors and their crosstalk driving fate commitment, as reviewed by Lee et al. [94].

**Figure 3:**
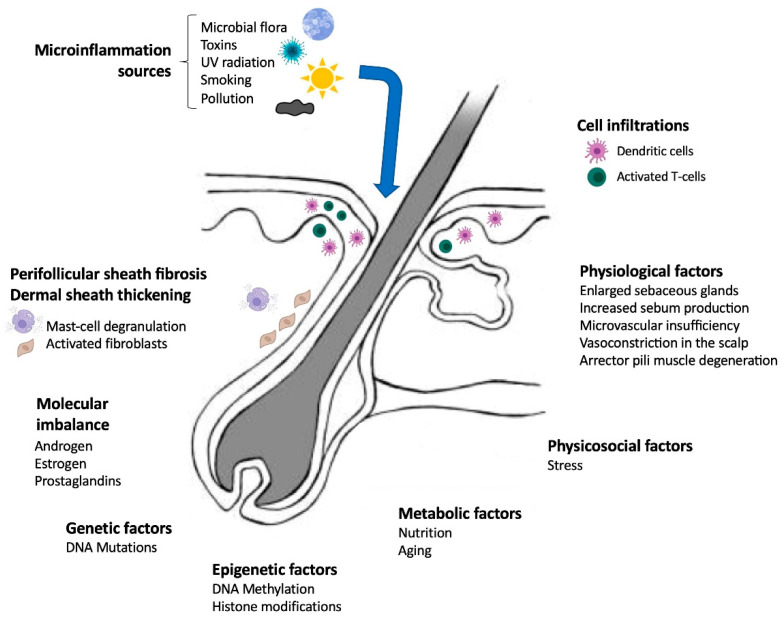
Numerous factors that contribute to the pathogenesis of androgenetic alopecia. All factors are interdependent and contribute to the formation of complex feedback loops. Individuals predisposed by inherited genetic mutations depict an increased sensitivity to DHT and a higher activity of 5α-reductase type 2 enzyme, driving downstream transcriptional dysregulations. Furthermore, environmental factors (pollution and UV radiation) and lifestyle-related factors (smoking, exercising, nutrition) potentially trigger epigenetic modifications that affect gene expression and result in protein imbalances (e.g., androgens, prostaglandins, cytokines). These protein imbalances alter cellular functions and signaling, causing, for example, the infiltration of immune system cells, dermal sheath thickening, perifollicular fibrosis, and other physiological factors. Sources of microinflammation in the infundibulum, including microbial flora, toxins, UV radiation, smoking, and pollution, maintain an altered signaling environment.

Other signaling pathways contributing to the pathogenesis of AGA have been suggested, as depicted in Figure 3. Early research using immunohistochemical analysis of alopecia biopsies demonstrated infiltration of activated T cells and dendritic cells in the lower portions of the follicular infundibulum [95]. Furthermore, researchers have found evidence of infiltration of inflammatory cells into the bulge, where the HFSC reside, as well as fibrosis in the perifollicular sheath [95]. These results pinpoint to a modest degree of chronic inflammation in the lowermost part of the infundibulum, potentially triggering the secretion of soluble factors that may directly or indirectly affect the cyclic stimulation of HFSCs [96]. Moreover, perifollicular infiltration of lymphocytes, histocytes, and mast cells has also been reported [97]. Evidence has demonstrated a role of inflammation in the pathogenesis of AGA at the clinical, histological, and transcriptomic levels [98,99,100,101]. The term “microinflammation” was adopted because, compared to scarring alopecias, inflammation in AGA is slow and subtle [102]. Evidence sustaining that this “microinflammation” plays an important role in AGA is the fact that drugs with anti-inflammatory effects, for example, minoxidil, can have a therapeutic effect. Several other factors can initiate microinflammation near the infundibulum, for example, microbial flora, toxins, oxidative stress, UV radiation, smoking, pollution, nutrition, metabolic syndrome, and aging [103,104,105]. However, the connection between androgens and microinflammation in AGA as well as the cell subtypes involved and their crosstalk remain unclear.

Although DHT is recognized as a significant contributor to AGA pathogenesis, DHT is only part of a complex orchestration of cascading and interacting events (Figure 3). Inherited DNA variants (genetic factors) increase a person’s predisposition to developing AGA; however, environmental factors (pollution and UV radiation), lifestyle habits (smoking, exercising, nutrition), and aging potentially influence gene transcription through epigenetic modifications [106,107,108]. Alterations in gene transcription (genetic or epigenetic) potentially translate into protein imbalances (e.g., androgens, prostaglandins, cytokines) that impact cellular functions and signaling [109,110,111]. Alterations in cellular signaling result in the infiltration of activated T cells and dendritic cells into the HF [95]. Moreover, disrupted signaling also results in an excessive collagen deposition, likely by dermal fibroblasts, producing dermal sheath thickening and perifollicular fibrosis [112,113]. It has been proposed that this excessive extracellular matrix deposition reduces oxygen and nutrient supply and interferes with signaling and removal of waste products, contributing to HF miniaturization [114]. All these factors are interdependent and synergistic, and they drive the emergence and progression of AGA.

To date, there are only two FDA-approved drugs for treating AGA: oral finasteride (1 mg/day) and topical minoxidil (2% and 5%). Finasteride is an inhibitor of the 5α-reductase type 2 enzyme, the main 5α-reductase in HFs. The rationale behind finasteride use in AGA is the absence of hair loss in individuals with congenital type-2 5α-reductase deficiency and the high levels of DHT and 5α-reductase activity in balding DP cells [115]. Another 5α-reductase inhibitor is dutasteride, which works on both type 1 and type 2 enzymes with a potency 100 and 3 times higher than finasteride, respectively [116]. Long-term treatments with finasteride or dutasteride have demonstrated hair regrowth and reversal of miniaturization in AGA (reviewed in [117,118]). However, finasteride is contraindicated in pregnant women due to risks of feminizing effects on male fetuses. The other popular FDA-approved AGA drug is minoxidil. Minoxidil is a potent activator of ATP-sensitive potassium channels, and it was originally used as a vasodilator to treat hypertension; however, an observed side effect was scalp hair growth [119]. In HFs, minoxidil is converted to its active form, minoxidil sulphate, by the SULT1A1 sulfotransferase enzyme found in the ORS [120,121,122]. Individuals with loss-of-function mutations in the *SULT1A1* gene have been referred to as non-responders to minoxidil [123].

Mounting research in vitro and in vivo have demonstrated that minoxidil prolongs the anagen phase promoting hair growth, induces resting follicles to grow, and decreases shedding [124,125,126,127]. Using cultured DP cells, researchers demonstrated that minoxidil induced an upregulation of *VEGF* at both transcriptomic and proteomic levels, suggesting that in vivo, minoxidil may induce angiogenesis through the expression of VEGF [128]. Minoxidil also demonstrated activation of the WNT/β-catenin pathway, involved in hair cycling and regeneration [129]. Moreover, in vitro studies using HFs from human scalp biopsies showed that minoxidil had a cytoprotective effect and increased the expression of prostaglandin PGE2 [130]. Prostaglandins have gained importance in studying AGA since their expression levels are dynamic during HF cycling, suggesting their regulatory roles. Furthermore, balding scalp samples depict high levels of PGD2 and low levels of PGE2 compared to non-balding zones [131]. Thus, prostaglandin pathways have become interesting research targets.

The continued use of minoxidil manages to arrest hair loss in at least half of the cases; however, only a small percentage experience moderate hair regrowth [132]. Unfortunately, the efficacy of minoxidil is variable and temporary. It is hard to predict its success since the mechanism of action in HF cells is not completely understood. Thus, researchers are working on developing novel drugs by targeting signaling pathways known to be involved in hair regeneration. Some of these strategies include the stimulation of WNT/β-catenin and the inhibition of BMP. The central role of WNT/β-catenin in hair morphogenesis, hair cycling, and regeneration [133,134] has led scientists to search for activators of this signaling pathway as potential therapeutic targets for AGA. For example, Lee et al. demonstrated that valproic acid induced hair regrowth as efficiently as minoxidil by activating WNT/β-catenin pathway in cultured human DP cells and in mice wounds [135]. Another strategy adopted by researchers is interfering with downstream targets or inhibiting proteins found to be increased either in late anagen or in alopecia patients. For example, Ryu et al. induced alopecia using CXXC5 knock-out mice [136]. Interestingly, results demonstrated that both DHT and PGD2 increase the expression of prostaglandin D_2_ synthase (PTGDS) and CXXC5, a zinc-finger protein that negatively regulates the WNT/β-catenin pathway [136]. Therefore, inhibition of negative regulators of WNT/β-catenin pathway is also a potential therapeutic approach for AGA treatment.

Novel treatment options that have recently been proposed include clascoterone, the family of JAK inhibitors, and prostaglandin analogs. Clascoterone was the first topical antiandrogen drug approved by the FDA in 2020 for treating hormonal acne. Due to its molecular similarity with DHT, clascoterone antagonizes ARs on DP cells, inhibiting AGA pathogenesis [137]. Clinical trials are ongoing to evaluate the efficacy and safety of clascoterone solution in AGA. Janus-kinase (JAK) inhibitors, including ruxolitinib, baricitinib, tofacitinib, and upadacitinib, have been proposed for the treatment of alopecia areata [138,139]. Furthermore, baricitinib was recently approved by the FDA (2022) for this pathogenesis. Alopecia areata is an autoimmune disease resulting in T cells damaging the hair follicle. JAK signaling in T cells causes them to release interferon-gamma, generating a positive feedback loop [140]. JAK inhibitors reverse this loop and allow hair regrowth in alopecia areata patients. However, the efficacy of JAK inhibitors in AGA is unclear and more studies are needed. Prostaglandin analogs, for example, latanoprost, were used to treat glaucoma patients and they experienced eyebrow and eyelash hypertrichosis side effects [141]. Researchers have demonstrated that latanoprost prolongs the anagen phase [142,143]. Nevertheless, more experiments and clinical trials are needed.

## 5. Transcription Profiling Studies of AGA

Transcription profiling is a popular functional genomics assay that involves the quantification of gene expression at the level of transcription (mRNA). Studies addressing genome-wide transcription in human hair follicles have been performed using microarrays and next-generation sequencing technologies, with the latter method depicting a higher resolution. These studies have focused mainly on finding the transcriptional differences in HFs obtained from the balding and non-balding regions of AGA patients and comparing AGA follicles to those of healthy controls. Assays using HFs obtained through plucking, follicular unit extraction (FUE), and skin biopsies have been performed. Through a comprehensive literature search, we found 9 studies involving human HGs [144,145,146,147,148,149,150,151]. In most studies (67%), the complete HF was used to obtain mRNA samples. In the remaining studies, the bulge and DP regions were isolated to obtain profiles of stem and progenitor cells. In 6 studies (67%), only AGA patients were included and paired comparisons were performed between balding and non-balding HF profiles. Table 1 lists the transcriptional studies found involving human HFs to describe AGA pathology. As observed, the number of studies performed using fresh human HFs as well as their sample sizes is small. Even so, researchers found modest numbers of differentially expressed genes (DEGs) between the balding regions (frontal and/or vertex) compared to non-balding (occipital) ones. Moreover, DEGs found are not limited to mRNAs; instead, miRNA and long non-coding RNAs have also been suggested. Interestingly, similar gene sets were overrepresented in AGA or balding regions in most of the studies, for example, inflammatory and immune responses, androgen signaling, and fibrosis. Similarly, common underrepresented gene sets included hair cycling, keratinocyte proliferation, WNT/β-catenin, TGF-β, BMP, and vitamin D biosynthesis.

The potential reasons for the small number of genome-wide transcription studies performed relies on several challenges. One of them is the difficulty in obtaining intact human HFs. Complete HFs including the DP can only be obtained through invasive techniques such as FUE or scalp biopsies. Both extraction methods are performed under local anesthesia. Another challenge faced when performing human HF transcriptional studies is the inability to predict and control the hair cycle stage of the extracted HFs. This problem introduces a high variability in the transcriptional profiles being compared since it has been demonstrated that HFs differ greatly depending on the hair cycle stage [152]. Working with animal HFs, for example, rats or mice, is an alternative to circumvent the hair cycle stage challenge since their HFs cycle synchronously in waves [55]. The last challenge is the cellular heterogeneity underlying the HF. This heterogeneity cannot be addressed adequately using bulk RNA-seq since the expression values of the genes are averaged between all cell types.

Instead, single-cell RNA sequencing is recommended. In a pioneering study by Joost et al. [153], researchers performed single-cell RNA sequencing of 1422 cells obtained from murine telogen epidermis and found 25 distinct cell subpopulations of interfollicular and follicular epidermis. Moreover, the transcriptional heterogeneity found was elegantly explained by spatial signatures even though the clusters shared a common basal–epidermal gene module. Afterwards, researchers from the same laboratory systematically addressed the transcriptional profiles and spatial location of cell subpopulations found in murine full-thickness skin during anagen and telogen stages [154]. A total of 55,767 and 7601 single cells conforming testing and validation datasets, respectively, were sequenced, and 56 transcriptionally distinct cell types and cell states were found. Interestingly, 20 different subpopulations were found to be specific to anagen HF [154]. The human counterpart of these studies was performed by Takahashi and colleagues [155]. In this study, human skin samples discarded from hair transplant procedures were processed, and 22,000 cells from the anagen HFs of 5 patients were isolated and profiled. A total of 23 cell subpopulations were identified and depicted enrichment of known marker genes [155]. This work suffered from scarcity of samples and a low depth of coverage; however, it underlines the heterogeneity of human HF cells.

## 6. Genome-Wide Association Studies of AGA

While progress has been achieved in understanding the molecular mechanisms of androgen metabolism in the HF, the genetic predisposition remains poorly understood. It has been demonstrated through studies involving twins that AGA is an autosomal dominant disorder in males, where a heritability rate of 80% was found [156]. Furthermore, it has been well established that AGA inheritance follows a polygenic model, involving the effect of multiple independent genes. Polymorphisms of the *AR/EDA2R* locus on the X chromosome were the first risk factors found to be associated with AGA [157,158]. The polymorphisms of the *AR* gene included two short tandem repeats (GGC and CAG) and a single-nucleotide polymorphism (SNP) in exon 1 which is recognized by the restriction enzyme Stul. Interestingly, early research demonstrated that this SNP was present in 98% and 92% of young and old bald men, respectively; nevertheless, it was also found in 77% of non-bald men [157]. These results suggest that this SNP is essential for AGA in men; however, other susceptibility genes also contribute.

**Figure 4:**
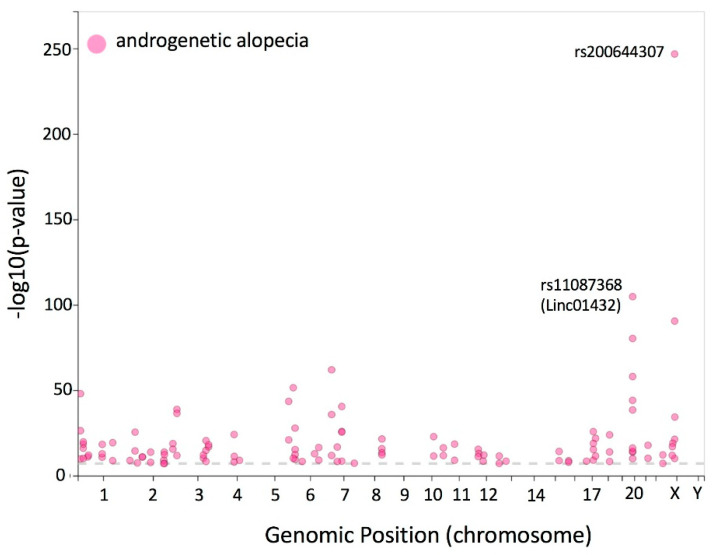
Manhattan plot depicting the association score as −log10 (*p*-value) corresponding to 119 significant variants related to androgenetic alopecia. Association scores and variants were retrieved from the GWAS catalog [159].

Genome-wide association studies (GWAS) have identified numerous genome-wide risk variants (typically SNPs) associated with AGA. To date, the GWAS catalog, a publicly available database of trait-associated SNPs, reports 119 variants significantly related to AGA [159]. These variants were derived from eight comprehensive studies published between 2008 and 2021. The most statistically significant variant is rs200644307, located in an intergenic region of chromosome X. Interestingly, the gene closest to this locus is *AR*, located at a distance of 500 kbs. Although the function of this locus has not been annotated, its location suggests that this might be a *cis*-regulatory element or enhancer that potentially regulates the transcription of the AR. Other statistically significant variants are found in *LINC01432*, a long intergenic non-protein coding gene located in chromosome 20. According to the GTeX portal, *LINC01432* is highly expressed in testis tissue, suggesting an association with male AGA [160]. Furthermore, *LINC01432* is located at approximately 330 kb downstream from *PAX1*, a transcription factor that has been previously reported to be correlated with AGA [161]. Other AGA susceptibility loci include androgen-dependent genes *EDA2R*, *HDAC4*, *SRD5A2*, and *FOXA2* as well as androgen-independent pathways *WNT10A*, *PAX1*, *MAPT*, *TARDBP*, *HDAC9*, *AUTS2*, *EBF1*, and *STBP1*. Figure 4 depicts a Manhattan plot of the 119 significant AGA variants found in the GWAS catalog. More information of each variant is included in Supplementary Table S1. Even though several AGA-associated loci have been identified through GWAS, several questions remain unanswered. For example, it is unclear if multiple SNPs in the same loci contribute to AGA risk. Moreover, the target genes of several variants are still unknown. Intriguingly, most risk variants are located in noncoding genomic regions and the precise biological mechanisms through which they contribute to AGA remain unclear.

## 7. Conclusions

The HF is a fascinating mini-organ that regenerates throughout adulthood in a cyclic mode mimicking embryonic development. To accomplish this, there is a tight spatial and temporal regulation of cell subpopulations achieved through autocrine and paracrine signaling. Importantly, alterations in these regulatory circuits are responsible of altered cycling and yield hair disorders such as AGA. Considering the many pathogenic mechanisms involved in the development of AGA, a multi-therapeutic approach is needed. Drugs that can regulate HF cycling, protect HF cells from autoimmune inflammatory-cell infiltrates, and control the expression of ARs and related enzymes represent important therapeutic approaches. However, there are still many gaps in our knowledge of HFs. Time-course profiling with single-cell resolution of all cell subpopulations of the HF is required for comprehensive characterization of the complex molecular changes occurring in HF cycling. Furthermore, the epigenomic profiles of HF cell subpopulations should also be addressed. Epigenetic modifications, for example, methylation and histone markers, have been demonstrated to play prominent roles in transcriptional regulation. Demethylation of promoters, potentially due to aging and environmental factors, may underlie the higher expression of ARs in balding scalp HFs. Thus, a multi-omics approach for the characterization of HF cell subpopulations during the hair cycle would greatly improve our understanding of the complex regulatory mechanisms underlying cycling. These will allow for the generation of more efficient, personalized and targeted therapeutic solutions.

## Figures and Tables

**Table 1:** Transcriptional studies performed using human HFs to describe AGA pathology. FUE: follicular unit extraction; DEGs: differentially expressed genes; NA: not available; * Unclear if gene sets are up- or downregulated since authors performed gene-set enrichment analysis using all DEGs.

Sample Size	Comparisons	Extraction Method	Number of DEGs in AGA	Upregulated Gene Sets in AGA	Down Regulated Gene Sets in AGA	Technology/ Platform	PMID/ Repository
5 male patients	HFs from bald vs. haired regions	FUE	250 (169 up, 81 down)	Immune response	Keratins	Affymetrix HG-U133A	22440736/GSE36169
20 patients, 10 healthy controls	Hair bulbs were used. Patient vertex vs. control vertex, patient vertex vs. patient occipital, patient occipital vs. control occipital, control occipital vs. control vertex	FUE	1339 (692 up, 647 down) (Patient vertex vs. Control vertex + occipital)	Lipid synthesis, electron carrier activity, metabolites, and energy	Keratin, epidermis development, cell cycle, hair follicle morphogenesis	Illumina HiSeq	27239811/NA
14 young (<35) patients (premature AGA) and 14 healthy controls	Scalp vertex, patients vs. controls	Biopsy	333 (184 up, 149 down)	Immune and inflammatory responses	WNT/β-catenin, TGF-β, BMP, and vitamin D biosynthesis	Agilent Whole Human Genome Oligo Microarrays	28403520/GSE90594
3 male patients	Scalp biopsies from bald and haired portions	Biopsy	431 (258 up, 173 down)	Cell proliferation, apoptosis, MAPK signaling, WNT signaling, EMT, TGF-β, GDF, and Activin *		Affymetrix Human Genome U952A2 microarray chips	28263792/NA
6 male patients	HFs from frontotemporal, vertex, and occipital regions	plucking	744 (336 up, 308 down)	Apoptosis, RNA methylation, ion channels	Cholinergic receptors, keratin production	Agilent Whole Human Genome Oligo Microarrays	28266729/GSE78722
10 male patients	Paired HFs from the balding and haired regions	Biopsy	2143 lncRNAs (770 up, 1373 down)	Metabolic process, T-cell receptor signaling	Developmental process, nervous system development, Hedgehog signaling	Human lncRNA Expression Microarray	28419572/GSE84839
24 male patients	Paired HFs from frontal vs. occipital scalp	plucking	143 miRNAs and 2836 mRNAs	Ceramide biosynthesis, GADD45 signaling *		Illumina HT12 Gene expression BeadChip, Affymetrix miRNA 4.0 Array	30009830/EGAS00001002832
12 male patients, 12 healthy controls	Bulge HFSCs vs. DP, AGA vs. controls, balding vs. non-balding areas	FUE	NA	Inflammation, stress, fibrosis	NA	Illumina HiSeq 2500	29122575/GSE101451
10 male patients	Paired HFs from the vertex and occipital scalp of 10 male patients with AGA	FUE	mRNAs: 308 up, 198 down; miRNAs: 35 up, 20 down; lncRNAs: 53 up, 74 down	HIF-1 signaling pathway	WNT and Hippo signaling pathways	Illumina HiSeqX Ten	35862273/NA
10 male patients	Paired HFs from the frontal and occipital scalp of 10 male patients with AGA	NA	NA	NA	NA	Illumina HiSeq 2500	NA/GSE212301

## Data Availability

Data sharing is not applicable to this article as no datasets were generated or analyzed during the current study.

## Supplementary Materials

The following supporting information can be downloaded at: https://www.mdpi.com/article/10.3390/ijms25052542/s1.
